# Correction: Reco, M.d.O.N.; Soares-Marangoni, D.A. Randomized Controlled Trial Protocol on the Effects of a Sensory Motor Intervention Associated with Kangaroo Skin-to-Skin Contact in Preterm Newborns. *Int. J. Environ. Res. Public Health* 2024, *21*, 538

**DOI:** 10.3390/ijerph21081028

**Published:** 2024-08-05

**Authors:** Mariane de Oliveira Nunes Reco, Daniele Almeida Soares-Marangoni

**Affiliations:** 1 Graduate Program in Health and Development, Faculty of Medicine, Federal University of Mato Grosso do Sul, Campo Grande 79070-900, Brazil; mariane.reco@ebserh.gov.br; 2 Graduate Program in Movement Sciences, Institute of Health, Federal University of Mato Grosso do Sul, Campo Grande 79070-900, Brazil

## Error in Figure

In the original publication [1], there was a mistake in Figure 2 as published. The whole flow diagram is incorrect as it was replaced by a figure from another study of our group. The corrected Figure 2 appears below. The authors apologize for any inconvenience caused and state that the scientific conclusions are unaffected. The original publication has also been updated.

## Figures and Tables

**Figure 2:**
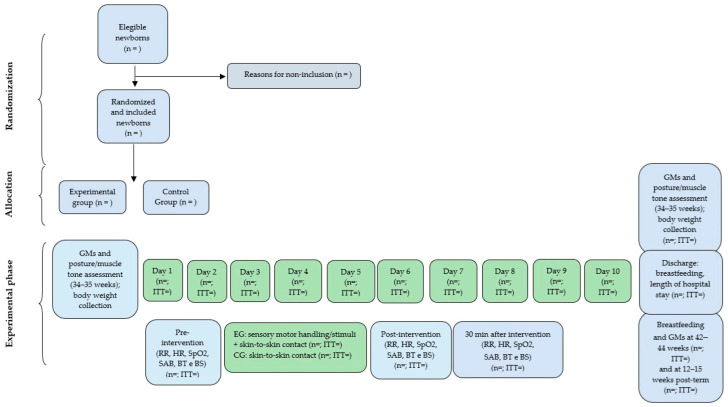
Flow diagram that will be used in the study. GMs: general movements, EG: experimental group, CG: control group, RR: respiratory rate, HC: heart rate, SpO2: oxygen saturation, SAB: Silverman–Andersen Bulletin, BS: behavioral state, BT: body temperature, ITT: intention-to-treat analysis.
